# RELATIONSHIP BETWEEN POST-STROKE TRUNK FUNCTION AND BRAIN LESION LOCATIONS: A SUPPORT VECTOR REGRESSION LESION-SYMPTOM MAPPING STUDY

**DOI:** 10.2340/jrm.v57.42782

**Published:** 2025-05-27

**Authors:** Keita NITTO, Hiroaki ABE, Yuka HASHIMOTO, Yutaro YABUKI, Mayu ARAI, Ryo SATO

**Affiliations:** 1 Department of Rehabilitation Medicine, Minami Tohoku Fukushima Hospital, Fukushima; 2 Department of Physical Therapy, Fukushima Medical University School of Health Sciences, Fukushima, Japan

**Keywords:** stroke, trunk function, Trunk Control Test, support vector regression lesion-symptom mapping

## Abstract

**Objective:**

This study aimed to investigate the relationship between brain lesions and trunk function impairment in stroke patients.

**Design:**

Retrospective cohort study.

**Subjects/Patients:**

One hundred fifty-six first-time stroke patients admitted for rehabilitation between August 2021 and October 2023.

**Methods:**

Trunk function was assessed using the Trunk Control Test. Brain lesions were detected using magnetic resonance imaging scans. Support vector regression lesion-symptom mapping was used to identify brain lesions associated with trunk function on admission and discharge, adjusted for lesion volume, age, and lower limb motor impairment.

**Results:**

After adjusting for age, admission trunk function was linked to lesions in the right corticospinal tract, superior longitudinal fasciculus, superior thalamic radiation, and putamen. Further adjustment for lower limb motor impairment revealed associations not only with all aforementioned regions, but also with lesions in the right supplementary motor area and premotor cortex. For trunk function on discharge, no suprathreshold regions were found.

**Conclusion:**

Early post-stroke trunk control impairment was associated with lesions in the right hemisphere, which is involved in motor function, motor control, and sensory integration. These findings provide insights into trunk dysfunction mechanisms, and suggest that targeted rehabilitation could improve trunk control and independence in daily activities for stroke patients.

## LAY ABSTRACT

Stroke patients often experience difficulty controlling trunk movements, which makes sitting, standing, and walking challenging. The present study investigated the lesion locations responsible for trunk function in 156 stroke patients. Using MRI, we found that damage to specific areas in the right brain hemisphere, including those involved in motor control and sensory functions, was linked to impaired trunk function in the early post-stroke period. Understanding which lesion locations are crucial for trunk function can provide valuable insights into the underlying mechanisms of trunk impairment. By elucidating the relationship between brain lesions and trunk dysfunction, this study can be used to guide the development of targeted rehabilitation strategies to improve functional independence in stroke patients.

The trunk function serves as a central axis for physical activities such as sitting, standing, and walking, supporting postural stability and -improving the efficiency of limb movements. Many stroke patients experience impairment in trunk function (1), which influences sitting balance, walking, and activities of daily living (ADL) (2). In particular, early post-stroke trunk function is known to be associated with walking prognosis (3), highlighting the importance of evaluating trunk function in stroke rehabilitation.

Trunk function consists of various components, such as coordination, muscle function, sensory function, and balance (2). Although trunk function impairment is often observed in the early stages after stroke, residual deficits may persist even beyond 6 months after onset (1). Given the impact of trunk function on daily activities and mobility, its recovery is a crucial rehabilitation goal. While the efficacy of training to improve trunk function has been demonstrated, the treatment effects can vary (2). Previous studies have shown that limb motor paralysis after stroke is often accompanied by reduced trunk function (4). The trunk and lower limbs are functionally connected through the pelvis, and lower limb paralysis may impair trunk movements during activities such as rolling (5) and ambulation (6). Moreover, trunk dysfunction has been associated with factors extending beyond motor paralysis, including sensory deficits (7) and unilateral spatial neglect (USN) (8). Identifying the determinants of trunk function recovery and clarifying the associated lesion locations could contribute to understanding the pathophysiology of trunk impairment, and lead to provision of optimal rehabilitation interventions. While recent studies have revealed associations between brain lesion locations and various functional impairments (9–11), the specific relationship between lesion location and trunk dysfunc-tion remains to be elucidated. If the differences in the recovery process of trunk function and associated brain lesions can be clarified, this could lead to the provision of tailored rehabilitation interventions based on the patient’s pathology and the development of novel treatment methods. However, to the best of our knowledge, no studies have yet investigated the brain lesions associated with the severity of trunk function impairment after stroke.

The aim of the present study was to investigate the relationship between specific brain lesions and trunk function impairment severity in first-ever stroke patients, using magnetic resonance imaging (MRI) findings and clinical assessments, including trunk function, and applying the support vector regression lesion-symptom mapping (SVR-LSM) (12).

## Methods

### Participants

From 416 patients admitted to Minami Tohoku Fukushima Hospital with a diagnosis of cerebral infarction or cerebral haemorrhage in the supratentorial or infratentorial regions between August 2021 and October 2023, we identified 257 first-time stroke patients. Our institution is a regional hospital with acute care and subacute rehabilitation capabilities. This study included patients in the acute or subacute phase who were prescribed physiotherapy following admission. The diagnosis of stroke was made by physicians after computed tomography or MRI scans confirmed cerebral infarction or haemorrhage. We analysed 156 patients after excluding those who had died, experienced symptom worsening during hospitalization, had not undergone MRI, had not been independent in ADL prior to admission, had incomplete imaging data, or had serious complications affecting daily life (e.g., spinal cord disorders or fractures).

This study was conducted with approval from the Ethics Committee of Minami Tohoku Fukushima Hospital (approval number: 23-4).

### Functional assessment

Trunk function was evaluated using the Trunk Control Test (TCT). TCT is a well-established evaluation method with verified reliability and validity for assessing trunk function in stroke patients (13). The assessment consists of 4 items: (*i*) rolling to weak side, (*ii*) rolling to strong side, (*iii*) sitting up from a lying position, and (*iv*) balance in a sitting position. Each item is scored on a 3-level scale with values of 0, 12, or 25 points, with total scores ranging from 0–100 points.

Assessments were conducted on admission, discharge, and at monthly intervals, and for this study, we used the results from admission and discharge.

Additionally, we retrospectively examined medical records for age, type of stroke (cerebral haemorrhage or cerebral infarction), affected hemisphere, length of hospital stay, days from stroke onset to imaging, days from onset to initiation of physiotherapy intervention at our hospital, and clinical assessment items at admission and discharge, including Fugl–Meyer assessment for lower extremity (FMA-LE) (14, 15), Berg Balance Scale (16), Functional Independence Measure (17), and Stroke Impairment Assessment Set (SIAS) components (18) such as light touch sensation (SIAS-Touch), position sense (SIAS-Position), and visuospatial perception (SIAS-USN).

### Lesion analysis

The MRI equipment used in this study was a 1.5T (SIGNA -Explorer; GE Medical Systems, Chicago, IL, USA), with imaging parameters for T2-weighted imaging (echo time = 90ms, repetition time = 4,500 ms, flip angle = 160°, slice thickness = 5 mm, acquisition matrix = 320×224, image matrix = 512×512, field of view = 220 mm×220 mm), diffusion-weighted imaging (DWI) (echo time = 80 ms, repetition time = 5,000 ms, flip angle = 90°, slice thickness = 5 mm, acquisition matrix = 128×128, image matrix = 256×256, field of view = 220 mm×220 mm), and fluid attenuated inversion recovery (FLAIR) (echo time = 130 ms, repetition time = 10,000 ms, inversion time = 2,600 ms, flip angle = 110°, slice thickness = 5mm, acquisition matrix = 290×190, image matrix = 512×512, field of view = 220 mm×220 mm). Additionally, MRI scanning in this study was performed based on physicians’ clinical judgement.

MRIcron software (http://www.mricro.com/mricron) was used to identify lesions. DWI and FLAIR were pre-matched to each patient’s T2-weighted images by co-registration. The location and size of ischaemic lesions were determined using the DWI taken closest to the time of onset. For lesions, the location and size were determined using FLAIR, which depicts the haematoma itself as a low-signal area, with ischaemic changes in the surrounding tissue as a high-signal area. The intensity filter function of MRIcron was used to enhance lesion identification. Lesions were identified by comparing the signal intensity of the affected areas with that of normal tissue on the opposite side. Areas were classified as lesions if their signal intensity was at least 20% higher (≥ 120%) or more than 20% lower (< 80%) than that of normal tissue. If the lesion size differed across images taken at multiple time points, the image depicting the largest lesion was used for analysis.

Spatial standardization was performed using Statistical Parametric Mapping 12 (https://www.fil.ion.ucl.ac.uk/spm/software/spm12/). First, the T2 image was multiplied by the lesion image as a mask image to exclude lesion areas from the T2 image. The lesion-masked T2-weighted images were segmented and spatially standardized (segm-normalization) based on this information, and the lesion images were spatially standardized based on the same algorithm. The lesion images were next visually inspected for correct spatial standardization, and then further normalized to Montreal Neurologic Institute (MNI) space using the MNI 152 template. The number of MRI voxels associated with each stroke lesion was counted and calculated as the lesion volume. All image analyses, including preprocessing, were conducted by experienced analysts at a separate institution, who were blinded to clinical data and had access only to imaging data. Clinical data were provided to the analysts only after the image analyses were completed and locked, ensuring that no further modifications could be made based on clinical information.

To investigate the relationship between lesions and TCT, the SVR-LSM method was employed. We used the SVR-LSM analysis tool, a graphical user interface running in MATLAB (https://www.mathworks.com/products/matlab.html), to -identify lesion sites associated with trunk function impairment (12). SVR-LSM (19) is a multivariate analysis technique that applies machine learning theory to identify associations between damaged voxels and behaviour while considering all lesion voxels. The conventional method, known as voxel based lesion symptom mapping (VLSM), had a problem in which voxels adjacent to truly associated lesions were often incorrectly detected as associated lesions, despite not being directly related (20). Type 2 errors (false negatives) often occur as a result of correcting Type 1 errors (false positives), and false positives themselves are generated because statistical tests are repeated thousands to tens of thousands of times (21). Vascular anatomy inherently leads to imbalanced statistical power in VLSM analyses, as some regions naturally exhibit a higher lesion frequency while others show few-to-no lesions. This imbalance has caused difficulty in VLSM analyses and indicates the limitations of the previous employed statistical methods (22, 23). SVR-LSM is superior to VLSM when multiple brain regions are involved in a single behaviour (22, 24). This is because in VLSM, each voxel is analysed individually, and if multiple brain regions are involved in a particular behaviour their interactions and complex relationships may not be adequately captured. In contrast, SVR-LSM utilizes machine learning to more accurately capture the relationship between multiple brain regions, even when they work together to influence behaviour. That is, instead of modelling the relationship for each voxel, the relationship of the behaviour with the entire lesion map can be modelled using a nonlinear function (19). This essentially takes into account correlations between voxels, and allows for more accurate detection of relationships between lesions and behavioural symptoms. SVR models are designed to accurately predict the impact of brain lesions on behaviour, such as speech and motor abilities, using information concerning the location of the damage.

In the present study, only voxels that showed lesions in at least 10 participants were included in the final analysis. Voxels that showed lesion overlap in fewer than 10 participants were considered insufficiently represented and were therefore excluded from the SVR-LSM analysis. A total of 10,000 permutation tests were performed, and statistical maps were corrected on a voxel-by-voxel basis at the voxel-wise threshold (*p* < 0.005), with cluster-wise correction applied only to clusters exceeding the threshold (*p* < 0.05). We created a colour map representing the z-scores of statistically meaningful voxels exceeding the cluster threshold, and overlaid the lesions on the JHU DTI-based white-matter atlases, Harvard-Oxford Subcortical Structural Atlas, XTRACT HCP Probabilistic Tract Atlases, and Human Sensorimotor Tracts Labels, which are all included in Functional Magnetic Resonance Imaging of the Brain (FMRIB) Software Library (FSL) (http://www.fmrib.ox.ac.uk/fsl).

### Quantification of disconnections in tract bundles

Disconnectome maps were calculated by BCBtoolkit (http://www.toolkit.bcblab.com/) (25), demonstrating the inter-individual variability of tract reconstructions based on control/template data. The result indicates the probability of disconnection from 0% to 100% for a given lesion (26). We quantified the severity of the disconnection by measuring the probability of disconnected tract (27) using Tractotron software as part of the BCBtoolkit (25).

### Statistical analysis

In the SVR-LSM analysis, age, which has been shown in prior research (28) to be associated with trunk function, was added as a covariate. If significant lesions were identified, we examined the relationship between TCT and factors that potentially influence trunk function, including age, FMA-LE, and SIAS (Touch, Position, USN), using Spearman’s correlation coefficient. Furthermore, considering the neurological and functional connectivity between trunk and lower limb function, we conducted SVR-LSM analysis with FMA-LE as an additional covariate. The purpose of this additional analysis was to examine brain regions associated with trunk dysfunction in greater detail by excluding the influence of lower limb motor impairment, thereby distinguishing between direct neural control of the trunk and effects derived from lower limb dysfunction.

The statistical analysis was performed using EZR (Saitama Medical Center, Jichi Medical University, Saitama, Japan), a graphical user interface for R (R Foundation for Statistical Computing, Vienna, Austria), with a significance level of *p* < 0.05.

## Results

### Patient characteristics

**Fig. 1:**
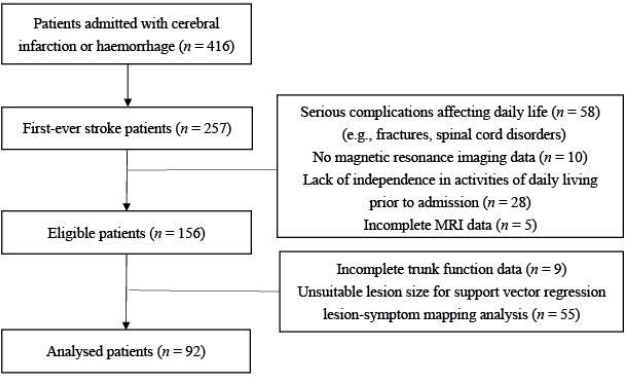
Flowchart of participant inclusion.

Of the 156 patients in this study, 9 were excluded due to missing TCT data, leaving 147 patients eligible for SVR-LSM analysis. From these 147 patients, a further 55 were excluded because their lesions were too small (lesion overlap with fewer than 10 participants) to meet the SVR-LSM analysis criteria (Fig. 1). The final analysis included 92 patients (54 males, 38 females) with age as a covariate (Table I). The patients’ median age was 73 years (interquartile range [IQR]: 60.75–81.25). There were 73 cases of cerebral infarction and 19 of cerebral haemorrhage, with 43 cases of damage in the right hemisphere and 49 in the left hemisphere. Of the 92 patients, there were 2 cases with lesions in the brainstem and cerebellum. The median hospital stay was 48.5 days (IQR: 18.5–101), the median time from stroke onset to MRI acquisition used in the analysis was 2 days (IQR: 0–4), and the median time from stroke onset to admission was 1 day (IQR: 1–14). Moreover, the median time from admission to initial physiotherapy was 0 days (IQR: 0–1), and the time from initial physiotherapy to the first TCT assessment was 1 day (IQR: 0–3). This study included both acute and subacute patients, with a median of 4 days (IQR: 1.75–17.25) from stroke onset to admission TCT assessment. Therefore, trunk function was evaluated early after stroke onset.

**Table I:** Demographic and clinical characteristics of participants

Factor	All analysed participants (*n *= 147)	Final analysed participants (*n *= 92)
Age (years), median (IQR)	74.0 (64–82)	73 (60.75–81.25)
Sex (male / female), *n*	85 / 62	54 / 38
Etiology (ischaemic / haemorrhagic), *n*	121 / 26	73 / 19
Stroke side (right / left), *n*	70 / 77	43 / 49
Length of hospital stay (days), median (IQR)	38.0 (16.5-93.5)	48.5 (18.5– 101)
Days from stroke onset to MRI scan for analysis (days), median (IQR)	1 (0–4)	2 (0–4)
Days from stroke onset to admission (days), median (IQR)	0 (0-11.5)	1 (1–14)
Days from admission to initial physiotherapy (days), median (IQR)	1 (0-1)	0 (0–1)
Days from initial physiotherapy to the admission TCT assessment (days), median (IQR)	1 (0-1)	1 (0–3)
Days from stroke onset to the admission TCT assessment (days), median (IQR)	4 (1-15.5)	4 (1.75-17.25)
TCT score, median (IQR)		
Admission	100 (61–100)	100 (61– 100)
Discharge	100 (100–100)	100 (100–100)
FMA-LE score, median (IQR)		
Admission	32.5 (26.5–34)	32 (24– 34)
Discharge	34 (31–34)	34 (31–34)
BBS score, median (IQR)		
Admission	48 (23.75-52)	48 (26.5–52)
Discharge	53 (45.5-56)	53 (45.5–56)
SIAS-Touch score, median (IQR)		
Admission	6 (4.5–6)	6 (4–6)
Discharge	6 (5–6)	6 (5–6)
SIAS-Position score, median (IQR)		
Admission	6 (4.5–6)	6 (4–6)
Discharge	6 (6–6)	6 (5–6)
SIAS-USN score, median (IQR)		
Admission	3 (3–3)	3 (3–3)
Discharge	3 (3–3)	3 (3–3)
FIM Total score, median (IQR)		
Admission	78 (50.5-90.5)	76 (49.5–90.25)
Discharge	119 (101.5-125)	120 (102.75–125)
FIM Motor score, median (IQR)		
Admission	48 (21-57)	48.5 (20–56.25)
Discharge	87 (72.5-90)	87 (75.75–90)
FIM Cognitive score, median (IQR)		
Admission	33 (25-35)	33 (25.75–35)
Discharge	35 (28.5–35)	35 (29–35)

IQR: interquartile range; TCT: Trunk Control Test; FMA-LE: Fugl-Meyer Assessment Lower Extremity; BBS: Berg Balance Scale; SIAS: Stroke Impairment Assessment Set; USN: unilateral spatial neglect (visio-spatil perception); FIM: Functional Independence Measure.

SVR-LSM analysis adjusted for age revealed significant lesion sites. The correlation analysis of TCT with age, FMA-LE, and SIAS (Touch, Position, USN) showed a moderately strong correlation with FMA-LE (*ρ* = 0.7315, *p* < 0.01), a moderate correlation with SIAS-USN (*ρ* = 0.6638, *p* < 0.01), and weak correlations with SIAS-Position (*ρ* = 0.5321, *p* < 0.01) and SIAS-Touch (*ρ = *0.4532, *p* < 0.01) (Table II). No correlation was found with age (*ρ = *–0.0409, *p* = 0.6).

**Table II:** Spearman’s correlations between trunk function and clinical measures on admission

Item	Age	FMA-LE	SIAS-Position	SIAS-Touch	SIAS-USN
Age	1				
FMA-LE	0.0813	1			
SIAS-Position	0.0451	0.4887*	1		
SIAS-Touch	0.169	0.476*	0.6867*	1	
SIAS-USN	–0.0033	0.6153*	0.606*	0.5302*	1
TCT	–0.0409	0.7315*	0.5321*	0.4532*	0.6638*

FMA-LE: Fugl-Meyer Assessment Lower Extremity; SIAS: Stroke Impairment Assessment Set; USN: unilateral spatial neglect (visuospatial perception); TCT: Trunk Control Test.

* *p*-values < 0.05 indicate statistical significance.

Based on these correlation results between TCT and each variable, we conducted another SVR-LSM analysis using FMA-LE as a covariate, with 87 patients included after excluding 6 due to missing FMA-LE data.

### Results of lesion analysis

**Fig. 2:**
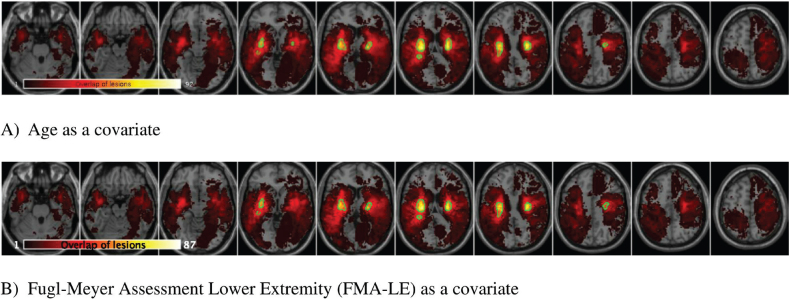
Lesion overlay maps of lesions included in support vector regression lesion-symptom mapping (SVR-LSM) analysis with: (A) age as a covariate (*n = *92), and (B) FMA-LE as a covariate (*n* = 87). The coloured bar represents the number of overlapping lesion areas, with red indicating fewer overlaps and yellow indicating more overlaps.

**Fig. 3:**
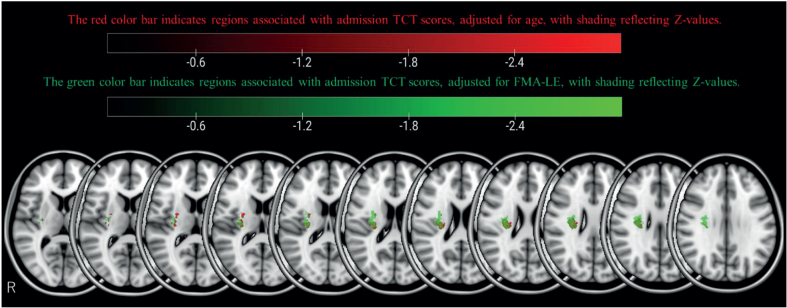
Support vector regression lesion-symptom mapping (SVR-LSM) results for Trunk Control Test (TCT) on admission, with age (red) and Fugl-Meyer Assessment Lower Extremity (FMA-LE) (green) as covariates. SVR-LSM results for the admission trunk control score are shown at voxelwise *p < *0.005 and clusterwise *p* < 0.05. The red area shows the results adjusted for age as a covariate, and the green area shows the results adjusted for the FMA-LE score.

Fig. 2 displays overlay images of lesions included in the SVR-LSM analysis, using the admission TCT scores and adjusting for age or FMA-LE as covariates. The results of the SVR-LSM analysis adjusted for both age and FMA-LE are presented in Fig. 3.

**Fig. 4:**
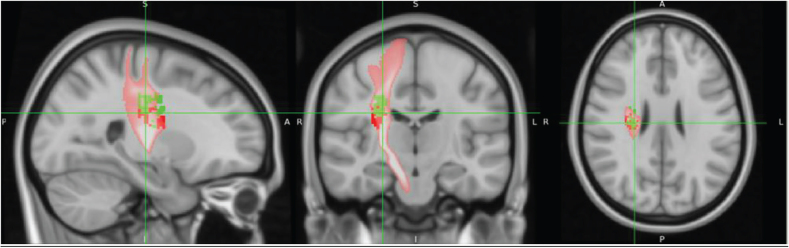
Identification of the lesion location in the corticospinal tract (CST). Regardless of whether age or Fugl-Meyer Assessment Lower Extremity (FMA-LE) was used for correction, the lesion overlapped with the trajectory of the CST. The anatomical location of the lesion was identified using the JHU White-Matter Tractography Atlas (Montreal Neurological Institute [MNI] coordinates: 25, –18, 26 when corrected for FMA-LE).

**Fig. 5:**
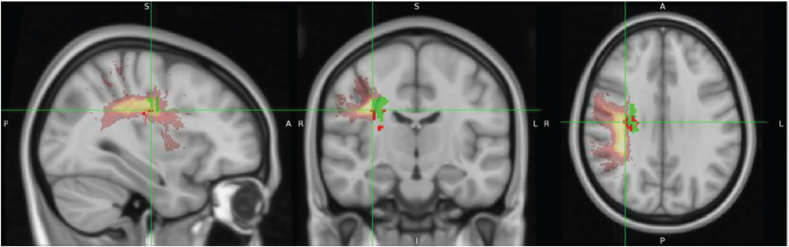
Identification of the lesion location in the superior longitudinal fasciculus (SLF) (corrected for age). The overlap between the lesion and the trajectory of the SLF is shown when corrected for age. The anatomical location of the lesion was identified using the JHU White-Matter Tractography Atlas (Montreal Neurological Institute (MNI) coordinates: 33, –15, 29).

SVR-LSM analysis of admission TCT scores, adjusted for age, revealed associations with the following right hemispheric regions: the corticospinal tract (CST) at the level of the superior corona radiata (Fig. 4), superior longitudinal fasciculus (SLF) (Fig. 5), superior thalamic radiation (STR) (Fig. 7), and putamen (Fig. 8).

**Fig. 6:**
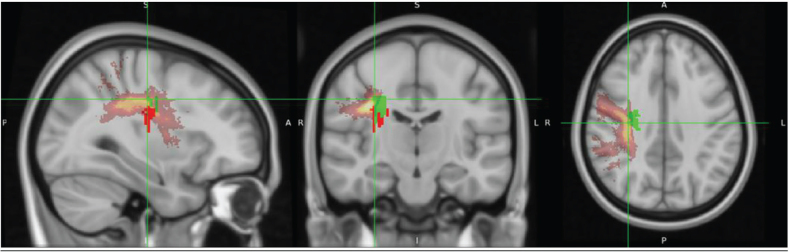
Identification of the lesion location in the superior longitudinal fasciculus (SLF) (corrected for Fugl-Meyer Assessment Lower Extremity [FMA-LE]). The overlap between the lesion and the trajectory of the SLF is shown when corrected for FMAL-E. The lesion was slightly more superior compared with the age-corrected case. The anatomical location of the lesion was identified using the JHU White-Matter Tractography Atlas (Montreal Neurological Institute [MNI] coordinates: 32, –17, 36).

**Fig. 7:**
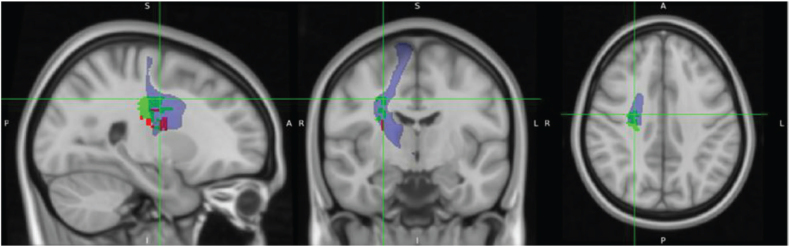
Identification of the lesion location in the superior thalamic radiation. The overlap between the lesion and the superior thalamic radiation is shown when corrected for age and Fugl-Meyer Assessment Lower Extremity (FMA-LE). The anatomical location of the lesion was identified using the XTRACT HCP Probabilistic Tract Atlases (Montreal Neurological Institute [MNI] coordinates: 25, –9, 37 when corrected for FMA-LE).

**Fig. 8:**
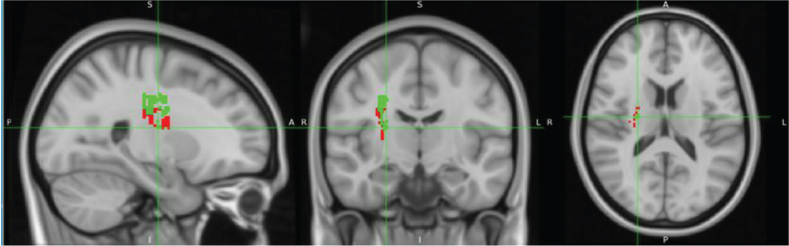
Identification of the lesion location in the putamen. The overlap between the lesion and the putamen is shown when corrected for age and Fugl-Meyer Assessment Lower Extremity (FMA-LE). The anatomical location of the lesion was identified using the Harvard-Oxford Subcortical Structural Atlas (Montreal Neurological Institute [MNI] coordinates: 25, –12, 15 when corrected for FMA-LE).

**Fig. 9:**
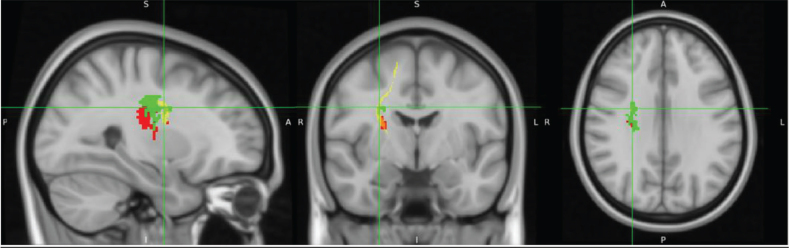
Identification of the lesion location in the supplementary motor area (SMA) and dorsal premotor cortex (PMd) (corrected only for Fugl-Meyer Assessment Lower Extremity [FMA-LE]). The overlap between the lesion and the fibres originating from the SMA and PMd is shown. The anatomical location of the lesion was identified using the Human Sensorimotor Tracts Labels (Montreal Neurological Institute [MNI] coordinates: 28, –5, 30).

**Fig. 10:**
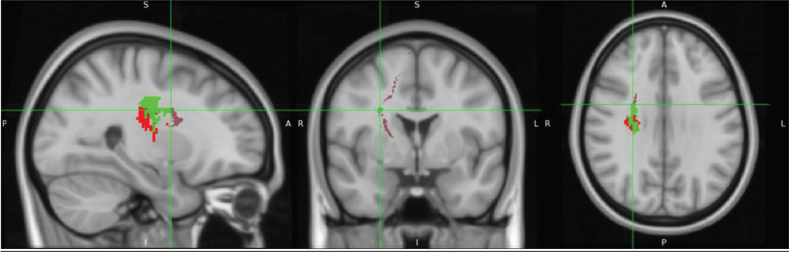
Identification of the lesion location in the presupplementary motor area (preSMA) (corrected only for Fugl-Meyer Assessment Lower Extremity [FMA-LE]). The overlap between the lesion and the fibres originating from the preSMA is shown. The anatomical location of the lesion was identified using the Human Sensorimotor Tracts Labels (Montreal Neurological Institute [MNI] coordinates: 27, –0.1, 29).

When admission TCT scores were adjusted for FMA-LE, the analysis demonstrated associations with the following right hemispheric structures: the CST at the level of the superior corona radiata (Fig. 5), SLF (Fig. 6), STR (Fig. 7), putamen (Fig. 8), dorsal premotor area (PMd), supplementary motor area (SMA) (Fig. 9), and presupplementary motor area (preSMA) (Fig. 10).

The lesion analyses adjusted for age or FMA-LE both revealed associations with the CST, SLF, STR, and putamen. However, the FMA-LE-adjusted analysis uniquely showed more extensive involvement of the STR and additional associations with the PMd, SMA, and preSMA.

No significant lesion associations were identified with discharge TCT scores.

### Results of disconnection analysis in tract bundles

**Fig. 11:**
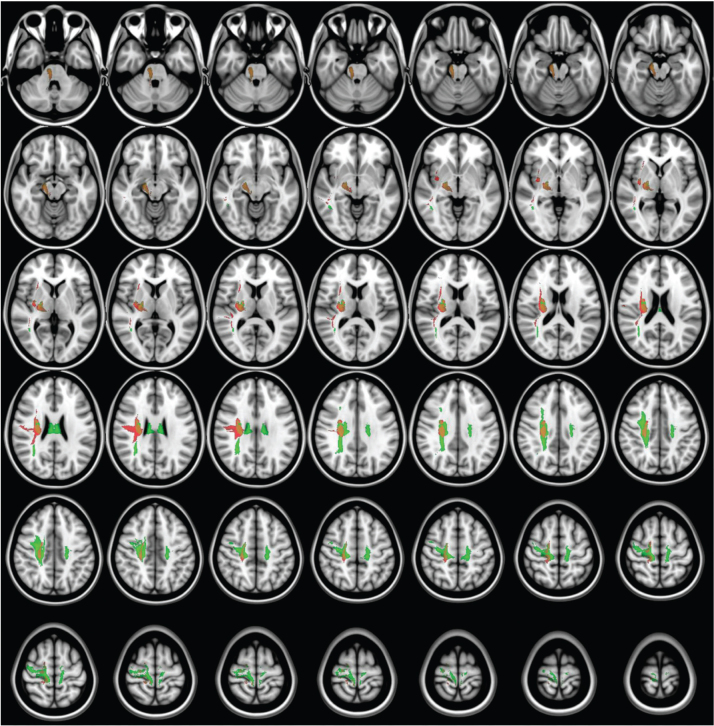
Disconnectome mapping of tracts with over 50% disruption. Disruption results for admission Trunk Control Test with age (red) and Fugl-Meyer Assessment Lower Extremity (green) as covariates.

Disconnectome mapping of tracts, with admission TCT adjusted for age or FMA-LE as covariates, is shown in Fig. 11, and the regions exhibiting over 50% disconnection in fibre bundles are summarized in Table III. After adjustment for age, disconnected fibre bundles were densely concentrated around the CST. When adjusted for FMA-LE, the disconnection area was observed to extend both anteriorly and posteriorly, as well as laterally. Under the age-adjusted condition, disconnections exceeding 50% were primarily observed in the right CST, Fronto-Insular Tract 5, and the CST (including SLF I, II, and III), with these disconnections -concentrated around the CST region. In contrast, under the FMA-LE adjusted condition, disconnections were found to extend over a broader range, affecting the corpus callosum, frontal CST, and other frontal white matter tracts, with the disconnections expanding anteriorly, posteriorly, and laterally from the CST region (Table III).

**Table III:** Tracts with over 50% structural disruption

Item	TCT covariate Age	TCT covariate FMA-LE
Anterior thalamic projections (Right)	47%	70%
Arcuate anterior segment (Right)	62%	24%
Arcuate long segment (Right)	66%	36%
Corpus callosum	28%	93%
Corticospinal (Right)	73%	85%
Frontal commissural	21%	81%
Frontal superior longitudinal (Right)	0%	73%
Fronto-insular tract 5 (Right)	69%	3%
Fronto-striatal (Right)	84%	89%
Handsup U tract (Right)	0%	52%
Pons (Right)	86%	92%
Superior longitudinal fasciculus I (Right)	0%	82%
Superior longitudinal fasciculus II (Right)	54%	98%
Superior longitudinal fasciculus III (Right)	86%	38%

TCT: Trunk Control Test; FMA-LE: Fugl-Meyer Assessment Lower Extremity.

## DISCUSSION

The aim of the present study was to investigate the relationship between trunk function impairment severity and specific lesion locations in stroke patients. Our findings revealed a striking right hemisphere dominance in trunk function control. These results support previous studies and suggest the important role of right hemisphere neural structures in trunk function (29). Both SVR-LSM analyses of admission TCT scores, with age and FMA-LE as covariates, demonstrated significant involvement of the CST, SLF, STR, and putamen in the right hemisphere. Furthermore, the SVR-LSM analysis with FMA-LE as the sole covariate revealed more extensive associations with the SMA and premotor cortex of the right hemisphere, as well as with the STR, the white matter tract connected to these cortical areas. In the disconnection map, after adjustment for age, disconnections were primarily concentrated around the CST. After adjustment for FMA-LE, disconnections extended more broadly, affecting multiple pathways connecting the frontal and parietal lobes, including additional frontal white matter tracts, with a wider distribution in the anterior, posterior, and lateral directions. These findings further support the notion that right hemisphere function is involved in maintaining trunk function after stroke, considering that the right hemisphere is involved in spatial cognition processing and postural control.

### Relationship between trunk function and brain lesions

SVR-LSM analysis with age as a covariate identified the CST and adjacent sensory regions as associated with admission TCT scores. Analyses with FMA-LE (an indicator of lower limb paresis) also showed an association with the CST, suggesting involvement of the anterior part of CST in trunk and proximal control. The CST is primarily thought to control the contralateral distal limb muscles. Age-adjusted analysis revealed significant associations between CST involvement and TCT scores. These associations remained significant even after controlling for lower limb paresis, suggesting that the observed relationship may specifically involve the anterior CST, which innervates trunk and proximal limb muscles. The CST includes ipsilateral descending pathways that are known to control proximal muscles, such as trunk muscles. Furthermore, a study investigating the relationship between trunk muscles and CST using transcranial magnetic stimulation reported that the magnitudes of ipsilateral and contralateral motor evoked potentials were approximately equal (30). These findings indicate that trunk muscles receive bilateral and contralateral innervation from the CST, which likely explains why the CST was identified as a lesion site relevant to trunk function even when FMA-LE scores were included as a covariate in the present study.

The association between the CST and admission trunk impairment severity suggests that targeted trunk muscle training may promote early recovery of trunk function. Previous research has demonstrated improvements in trunk function and ADL through trunk training focused on muscle function, such as isometric contraction of trunk muscles, selective voluntary movements of the upper and lower trunk, and electrical stimulation therapy (2). Future prospective studies should evaluate the treatment course of patients with CST lesions and impaired trunk function by investigating the effectiveness of trunk training and identifying specific factors that predict treatment response, in order to select the optimal rehabilitation strategy for each patient.

Additionally, the results of the present study also indicated an association between admission TCT and the SLF in the right hemisphere. The SLF is an associated fibre tract between the frontal lobe and the ipsilateral parietal and temporal regions (31), playing a crucial role in motor control, including information transmission for motor planning and integration of visuomotor information (32). Moreover, SLF I is specifically associated with movements that utilize spatial information, with recent studies highlighting its relationship with upper limb motor function (33). Furthermore, the right hemisphere SLF is thought to be a lesion site responsible for USN (34), which has been reported to be associated with reduced trunk function (8). The present study also demonstrated a correlation between admission TCT and SIAS-USN, indicating that USN may have contributed to the reduction in TCT scores.

Given that the SLF is a white matter tract connecting the frontal lobe to various other lesion locations, it cannot be concluded that its damage alone results in trunk function impairment. We also found lesions in the STR, which is a white matter tract that connects the posterior limb of the internal capsule to the ventral nuclear group of the thalamus and the pre- and post-central gyri (35). Furthermore, the STR is thought to be involved in sensory function. Our results demonstrated correlations between admission TCT scores and both SIAS-Touch and SIAS-Position, supporting previous research linking sensory impairment to reduced trunk function (7). Thus, the involvement of white matter tracts such as the SLF and STR, which establish networks across multiple lesion locations, suggests that impaired multisensory integration functions may indirectly influence trunk function. In particular, given that multisensory integration is predominantly mediated by the right hemisphere, our findings suggest that right hemisphere lesions may lead to more severe impairments in trunk function.

To further elucidate the detailed relationships between trunk function and the SLF, STR, and other sensory regions, studies should focus on patients with lesions in regions associated with various sensory integration functions, such as those with impairments in sensory function and visuospatial cognition. Additionally, using diffusion tensor imaging would be beneficial for investigating white matter tracts associated with sensory function in such patients and exploring their relationship with trunk function and clinical outcomes, including ADL performance. Identifying factors linking sensory and trunk func-tion could lead to future studies on rehabilitation treatments aimed at improving sensory function or integrating sensory and motor functions, providing clinically relevant insights.

This study’s SVR-LSM analysis revealed that trunk function was associated with the SMA and premotor cortex, only after adjusting for lower limb motor paresis. The SMA and premotor cortex are involved in anticipatory postural adjustments through the corticoreticular and reticulospinal pathways, and contribute to postural control and movement initiation (36). Furthermore, these regions are also responsible for the generation of motor programmes based on the integrated sensory information from the somatosensory and visual cortices, playing an important role in the perception of body orientation, movement, and object motion, as well as in the spatial awareness necessary for movement execution (36). Because the relationship was not observed when admission TCT scores were adjusted for age alone, the influence of motor paresis appears to be an extremely significant factor in trunk function impairment. However, the association of SMA and premotor cortex in the FMA-LE adjusted analysis suggests that damage to regions involved in anticipatory postural control and motor programme generation may lead to reduced trunk function, even in cases with mild motor paresis.

The putamen, as part of the basal ganglia network (37), has been implicated in impairments of trunk function, which are commonly observed in Parkinson’s disease (38). Similarly, in stroke patients, lesions in the putamen have been associated with vascular parkinsonism, suggesting that the latent effects of post-stroke parkinsonism might contribute to impairments in trunk function (39). Therefore, the observed association between putamen damage and trunk function impairment in the present study, independent of age or motor paresis severity, may be explained by subtle parkinsonism-like symptoms resulting from basal ganglia network damage.

### Study limitations

The SVR-LSM analysis in the present study was unable to identify brain lesions associated with discharge TCT scores or lesions locations involved in delayed trunk function recovery. This was because very few participants had persistent trunk function impairment on discharge. The current findings contrast with those of previous studies, which reported continued trunk function deficits beyond 6 months post-stroke (1). This difference may be attributed to the variation in the trunk function evaluation methods employed. While the TCT is a representative measure of post-stroke trunk function, it has been reported to have a ceiling effect (40). Therefore, using assessment tools recommended for more detailed evaluation of trunk function, such as the Trunk Impairment Scale (40), may contribute to investigations into the relationship between trunk function and brain lesions.

This study included both haemorrhagic and ischaemic stroke cases. In haemorrhagic stroke, favourable recovery may occur as the effects of oedema around the haematoma diminish through absorption. Therefore, differences in how recovery affects brain function may exist between stroke types. However, in our current dataset, all cases showed favourable recovery on discharge, suggesting that a ceiling effect in the TCT, or limitations in the assessment tool, may have influenced the results. Consequently, we were unable to identify brain regions associated with recovery, and associations were found only with admission data. Thus, we believe the inclusion of haemorrhagic stroke patients had only a limited effect on the results.

### Conclusion

In the present study, we conducted SVR-LSM analysis to investigate the relationship between trunk function and brain lesions in first-time stroke patients. When the admission TCT scores were adjusted for age, associations were found with the CST, SLF, STR, and putamen in the right hemisphere. Further associations were observed with the SMA and premotor cortex after adjusting for FMA-LE scores. These findings suggest that early post-stroke trunk function is affected by right hemisphere lesions, particularly those affecting not only motor pathways but also white matter tracts involv-ed in spatial cognition and multisensory integration.

## Trial details

This study was registered and conducted in the University Hospital Medical Information Network (UMIN) Clinical Trials Registry (Trial ID: UMIN000052087).
